# Case report: PRES associated with fruquintinib in a patient with metastatic colon cancer

**DOI:** 10.1007/s10072-023-06991-7

**Published:** 2023-08-15

**Authors:** Lu Wang, Zhaohao Zeng, Zhiqiang Wu

**Affiliations:** 1 Department of Neurology, Xiaogan Hospital Affiliated to Wuhan University of Science and Technology, Xiaogan, Hubei China; 2 Department of Neurology, Shenzhen People’s Hospital (The Second Clinical Medical College, Jinan University; The First Affiliated Hospital, Southern University of Science and Technology), 518001 Shenzhen, Guangdong China

**Keywords:** Posterior reversible encephalopathy syndrome (PRES), Fruquintinib, Metastatic colon cancer

## Abstract

Posterior reversible encephalopathy syndrome (PRES) is a rare, reversible neurological disease that is frequently associated with the use of targeted therapy agents. In this case study, we examine the development of posterior reversible encephalopathy syndrome (PRES) in a 44-year-old woman with metastatic colon cancer following 1 month of treatment with the vascular endothelial growth factor receptor (VEGFR) inhibitor, fruquintinib. The occurrence of PRES after 1 month of VEGFR inhibitor administration is a common phenomenon. However, it is noteworthy that this is the first reported case of PRES associated with fruquintinib. The patient’s neurological function improved upon discontinuing the drug for a week, but worsening was observed following a lower-dose fruquintinib treatment. This patient’s experience highlights the potential for neurological deterioration in those treated with fruquintinib, prompting physicians to consider the possibility of PRES. Notably, this may be the first reported case linking fruquintinib to the syndrome, underscoring the importance of recognizing the association between PRES and fruquintinib.

## Introduction

Posterior reversible encephalopathy syndrome (PRES) is a clinical and radiological syndrome that was initially described by Hinchey et al in 1996 [1]. It is typically characterized by reversible vasogenic edema that manifests symmetrically. In adults, there is a female predominance of cases even after exclusion of patients with eclampsia. The prevalence in the pediatric population has not been well established, although a study of 825 pediatric hospitalizations for PRES-associated conditions such as bone marrow transplant, hypertension, and connective tissue disorders found the rate of PRES to be 0.04% [2]. The patients developed a neurological syndrome dominated by seizures, headache, mental status alteration, and visual dysfunction [3]. Seizures are a prevalent symptom in PRES, occurring in approximately 81% of patients, typically presenting as generalized tonic-clonic episodes. Visual disturbances, including cortical blindness and visual neglect, were observed in up to 39% of patients in one study [4]. PRES is usually caused by severe hypertension, immunosuppressant use, renal failure, exposure to cytotoxic agents, and the use of molecular target-specific agents [5]. Fruquintinib is a novel vascular endothelial growth factor receptor (VEGFR) inhibitor that gained approval by the China Food and Drug Administration (CFDA) in 2018 for treating patients with metastatic colon cancer who had previously undergone standard anticancer therapies [6]. This report documents the case of a 44-year-old patient with metastatic colon cancer who underwent colorectal surgery, followed by fruquintinib treatment, and subsequently developed PRES. To the best of our knowledge, this is the first case report to describe an association between fruquintinib and PRES.

## Case report

**Fig. 1:**
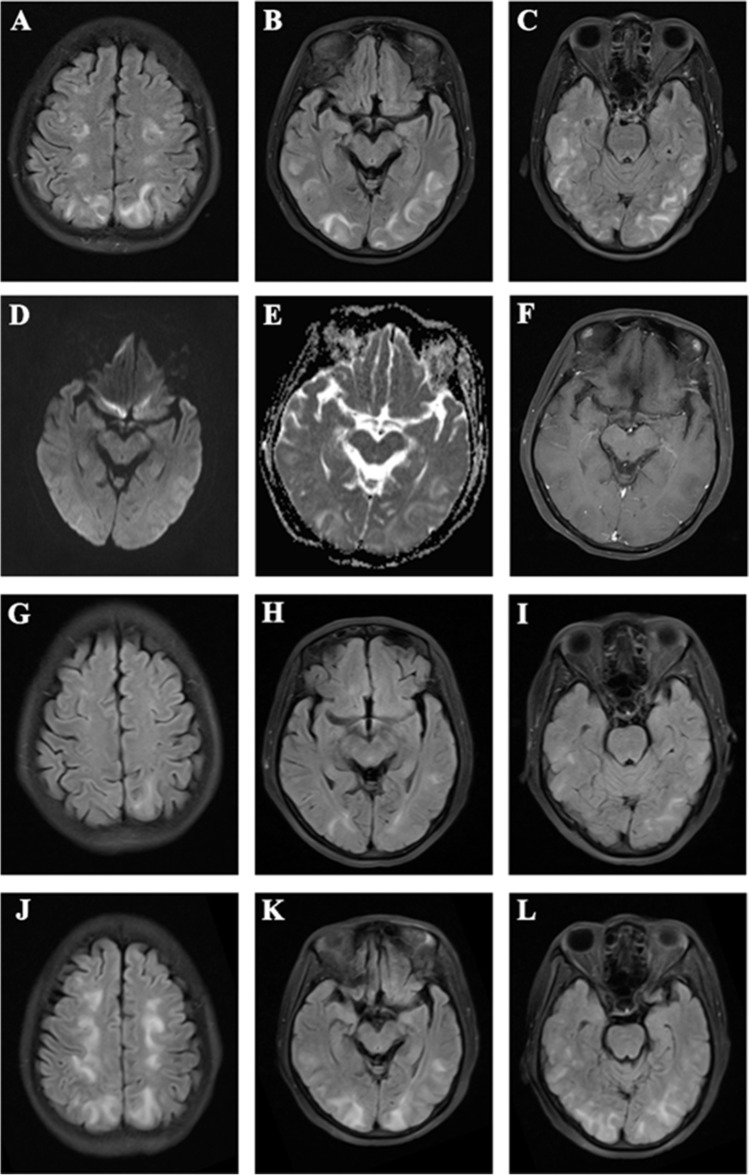
Brain MRI showing findings consistent with posterior reversible encephalopathy syndrome. **A**–**C** Increased symmetric signals on T2-Flair-weighted images of the parieto-occipital lobes. **D** and **E** Hyperintense lesions on DWI and increased ADC values. **F** Contrast-enhanced MRI scans show no enhanced signals. **G**–**I.** T2-Flair-weighted images after therapy. **J**–**L** Cerebral lesions recurred after a 4 mg dose of fruquintinib was restored

In August 2022, a 44-year-old woman with a history of right-sided colon cancer, which had been treated with radical resection, presented with visual disturbances and seizures. During the medical consultation, the patient experienced sudden limb convulsions, upward eye movement, and urinary incontinence, accompanied by impaired consciousness, lasting approximately 30 s. After administering a 5 mg intravenous injection of midazolam, the patient’s condition improved. Throughout the hospitalization, there were a total of two similar episodes with consistent clinical manifestations. Postoperative pathology showed moderately differentiated adenocarcinoma, sized 3.0 × 2.2 cm, with a positive margin and full-thickness infiltration involving the serosa. Subsequently, five cycles of the oxaliplatin and capecitabine regimen were given, but a severe allergic reaction ensued. On May 12, 2022, pelvic and abdominal cavity CT indicated intrahepatic nodules and abdominal cavity metastasis, along with massive ascites. When liver metastasis occurred, the patient was administered one cycle of bevacizumab and tegafur (a prodrug of 5-fluorouracil, authorized in the treatment of patients with colon cancer). By July 5, 2022, severe myelosuppression led to the adjustment of the chemotherapy plan, and the patient was started on fruquintinib and capecitabine. Fruquintinib (4 mg qd) appeared to be well tolerated by the patient after 3 weeks of treatment, with no significant adverse effects reported. However, the patient developed severe seizures after receiving fruquintinib (5 mg qd) for approximately 1 week. Although the patient had no history of hypertension, her blood pressure remained elevated during her hospitalization. Further MRI examination depicted a symmetric high-intensity area in the parieto-occipital lobes on T2-Flair-weighted images 10 days after fruquintinib treatment (Fig. 1A–C). There were hyperintense lesions on DWI and increased ADC values (Fig. 1D, E). Contrast-enhanced MRI scans show no enhanced signals (Fig. 1F). Normal blood vessels were reported in MRA. While tests for autoimmune encephalitis antibodies, central nervous system demyelination, and paraneoplastic syndrome were negative, serum antinuclear antibody (ANA) and cytoplasmic antineutrophil cytoplasmic antibody (c-ANCA) levels were within normal range. Blood tests revealed the following abnormalities: white blood cell count of 19.3 × 10^9/L (normal range: 3.5–9.5 g/L), platelet count of 61 × 10^9/L (normal range: 125–350 g/L), lactate dehydrogenase level of 347 U/L (normal range: 120–250 U/L), hemoglobin level of 103g/L (normal range: 115–150 g/L), and creatinine value of 50 umol/L (normal range: 41–73 umol/L). Additionally, the high-sensitivity C-reactive protein was elevated at 82.27 (normal range: 0–3 mg/L). During the course of the illness, the platelet count dropped to a minimum of 23 × 10^9/L, prompting the use of eltrombopag (an orally available thrombopoietin receptor agonist indicated for the treatment of immune thrombocytopenia) to promote platelet recovery. Cerebrospinal fluid (CSF) analysis showed elevated protein levels of 1032 mg/dL (normal range: 150–450 mg/dL) with normal glucose levels of 2.56 mmol/L (normal range: 2.5–4.4 mmol/L), and no oligoclonal bands were detected.

The patient had a history of hypothyroidism and was treated with unimethylate. During the hospitalization, the patient developed a urinary tract infection. The urine culture revealed the presence of multidrug-resistant *Escherichia coli*. The patient showed improvement after receiving treatment with the sensitive antibiotic meropenem, administered at a dose of 1 g every 8 h to combat the infection. During hospitalization, the patient was consistently taking sodium valproate solution to control epileptic seizures, along with controlled-release nifedipine tablets to manage blood pressure. In addition, she was administered 20 mg of intravenous dexamethasone and 100 ml of mannitol for 3 days, following which she gradually began to recover neurologically. Interestingly, during the use of steroids, the patient experienced visual hallucinations, reporting the perception of seeing chicken claws and noodles moving in front of their eyes. The duration of these symptoms was approximately 1 h, and they disappeared after discontinuing the steroid medication. On subsequent head MRI, the previously detected lesions had mostly faded away (Fig. 1G–I). Given the positive response of colon cancer to treatment with fruquintinib, which continued at a dose of 4 mg qd from August 19, 2022, the cerebral lesions resurfaced 3 days later (Fig. 1J–L). Based on the patient’s clinical presentation and neuroimaging findings, a new diagnosis of medication-induced reversible posterior leukoencephalopathy syndrome due to the use of fruquintinib was made. The patient’s neurological deficits improved after discontinuing fruquintinib, and the patient was monitored for 1 week.

## Discussion

We present a case of reversible posterior leukoencephalopathy syndrome that arose due to the administration of fruquintinib, and an MRI scan indicated vasogenic edema in the brain hemisphere. Notably, there are no universally accepted diagnostic criteria for PRES. Instead, a PRES diagnosis often relies on a combination of clinical presentation and radiographic abnormalities [7]. In a recent review of the published cases, Eiichi et al. [8] have reported a case of PRES after a variety of combined chemotherapies containing bevacizumab for metastatic colon cancer. Taimoor et al. [9] reported a case of oxaliplatin-induced PRES in a 23-year-old patient. While the incidence of PRES associated with targeted drugs is uncommon, there have been no prior reports linking fruquintinib to the syndrome. Notably, hypertension, proteinuria, and hand-foot skin reactions were some of the frequently observed adverse effects in patients receiving fruquintinib in previous studies [10]. Our patient had no known history of hypertension, and the raised blood pressure detected during admission could be attributed to the side effects of fruquintinib.

When presented with seizures, acute stroke, meningitis, encephalitis, or sinus thrombosis, it is essential to exclude these conditions in the initial diagnostic work-up. Furthermore, MRI scans can be useful in distinguishing other pathological conditions that exhibit similar clinical features to PRES. In our patient, a history of metastatic colon cancer, along with MRI findings indicative of symmetric vasogenic edema in the subcortical white matter of the parieto-occipital lobes, raised suspicion for PRES. The rapid neurological recovery observed in the patient and the consequent reversibility of the lesions detected in the MRI scan confirmed the diagnosis of PRES. Despite being commonly associated with chemotherapeutic agents, the exact pathophysiology of PRES is still not entirely understood. The breakdown of the blood-brain barrier is believed to be the result of hypertension and endothelial injury, as per accepted theories [11]. The most useful MRI sequences for detecting vasogenic edema are typically T2-weighted and T2 FLAIR (fluid-attenuated inversion recovery) sequences. Fruquintinib, an inhibitor of vascular endothelial growth factor, frequently induces adverse reactions including increased blood pressure, diarrhea, and skin manifestations. PRES is categorized as a rare adverse reaction, and no cases have been reported thus far. In our patient, the neuroradiological findings were consistent with the clinical manifestations observed, emphasizing the importance of imaging studies in aiding the diagnosis of PRES.

During the follow-up period, our patient received supportive care, as is usually recommended in the management of PRES. Treatment is aimed at controlling the underlying cause, such as hypertensive urgency or cytotoxic agents [12]. In the majority of PRES cases, clinical manifestations and MRI lesions are reversible. Early detection and intervention, such as treating hypertension or discontinuing cytotoxic medications, are important in achieving favorable clinical outcomes. Fortunately, the 44-year-old woman in our case report achieved complete recovery after discontinuing fruquintinib.

## Conclusion

In conclusion, our study highlights the significance of recognizing posterior reversible encephalopathy syndrome (PRES) as a relatively unfamiliar complication associated with the use of fruquintinib. Physicians should be particularly vigilant about the possibility of PRES in patients presenting with acute neurological deterioration.
